# Multiphasic Personality Assessment in a Case Series of Adolescent Patients with Suicidal Ideation and/or Attempts

**DOI:** 10.3390/children10111794

**Published:** 2023-11-08

**Authors:** Giulia Cossu, Arianna Vecchio, Marika Orlandi, Erica Casini, Renato Borgatti, Martina Maria Mensi, The Mondino Foundation Suicidality Research Group

**Affiliations:** 1 Department of Brain and Behavioural Sciences, University of Pavia, 27100 Pavia, Italy; 2 Child Neurology and Psychiatry Unit, IRCCS Mondino Foundation, 27100 Pavia, Italy

**Keywords:** adolescence, suicide attempt, suicide ideation, assessment, personality

## Abstract

Suicide is an important public health issue. To examine the differences in personality characteristics between a group of adolescents with suicidal ideation (SI) and a group with a history of suicidal attempts (SA), we conducted a cross-sectional study. We enrolled 55 adolescents (51 females; 12–18 y.o.) who presented SI and/or SA. Using the Columbia Suicide Severity Rating Scale, we divided the sample into two groups: adolescents with SI and adolescents with SA. All participants filled in the Minnesota Multiphasic Personality Inventory—Adolescent (MMPI-A). Adolescents in the SA group had greater difficulties in social relations, risky behaviors, and more intense suicidal ideation compared to those in the SI group. Adolescents in the SA group scored higher in Omission, in the Lie Scale, the Conduct Problem Scale, the Less Aspirations Scale, the Repression Scale in the MMPI-A, and item 283 of the MAST compared to the other group. The results suggest that using the MMPI-A to assess certain features (e.g., tendency to lie, repression) may be helpful in identifying young people who are at high risk of suicide. However, further research is required to determine the effectiveness of using this instrument.

## 1. Introduction

Suicide is a rising phenomenon that affects individuals all around the world and is particularly prevalent in youth. According to research in 2020, suicide ranks as the second most common cause of death for those between the ages of 10 and 19 [1]. According to recent findings, social isolation brought on by the lockdown situation is one of the theories explaining the increased likelihood of suicidal thoughts during the COVID-19 epidemic [2,3,4]. In addition, suicide risk is correlated with several factors, including social isolation, as well as psychiatric, genetic, environmental, temperamental, and sociocultural aspects [5,6,7]. Several studies have documented an increased prevalence of suicidal attempts (SA) in relation to completed suicide among younger people compared to adults (ratio SA:S = 200:1), as well as in the female population compared to male peers of the same age [8]. These data derive from the hypothesis that women are more likely to be introspective and verbally disclose when they are uncomfortable. As a result, in these cases, the self-aggressive act would seem to have a communicative purpose, which is further supported by the fact that females are more likely to use non-violent methods. In contrast to women who mostly employ poisoning, especially from narcotics, men use blunt or gunshot wounds, hanging, and precipitation more commonly, which are more violent methods [9,10].

Major affective disorders are the psychiatric conditions most associated with suicide [10], particularly major depressive disorder [11,12]. An increased risk of suicide is also linked to psychotic symptoms [13,14], borderline personality disorder [15], and alcohol and substance abuse [16,17]. The assessment of suicide risk necessitates the consideration of previous suicide attempts [18], the existence of disadvantageous environmental situations [19], including the premature death of a parent [20], the suicide of a parent [21], and the presence of scholastic stressors [22], as these aspects constitute risk factors for a potential suicide attempt [23].

According to previous studies, suicidality and perfectionism seem to be related [24]. However, other stressful life events, such as being bullied or the existence of other personality characteristics, including neuroticism, anxiety, extraversion, and depression, appear to mitigate this factor [25]. As already established, there are several risk factors for suicide. Some of these have been the subject of more extensive research than others. These include personality traits, although prior studies advise assessing them in order to help physicians customize the most effective early intervention, especially for children and adolescents [26]. Several researchers found that certain personality traits and characteristics, such as borderline personality traits, seem to predispose people to suicide, neuroticism, psychoticism, and extraversion are other significant traits that are more closely linked to suicidality. Moreover, the literature highlighted that individuals who exhibit perfectionism, interpersonal dependence, novelty seeking, impulsivity, pessimism, nonconformity, low self-esteem, feelings of inferiority, hopelessness, and self-criticism are more likely to have suicidal thoughts and commit suicide [23,27,28,29,30,31,32,33]. According to a study by Beautrais and colleagues, there is a link between suicidal behavior and personality characteristics such as neuroticism and introversion, also expressed through withdrawal and social isolation [34].

The present study aimed to examine which personality features are more associated with suicidal ideation and behavior in the adolescent population and the differences between these two groups. We would expect significant differences between the two groups in MMPI-A subscales, in the validity subscales and in specific items of the D Scale.

## 2. Materials and Methods

### 2.1. Participants

**Figure 1:**
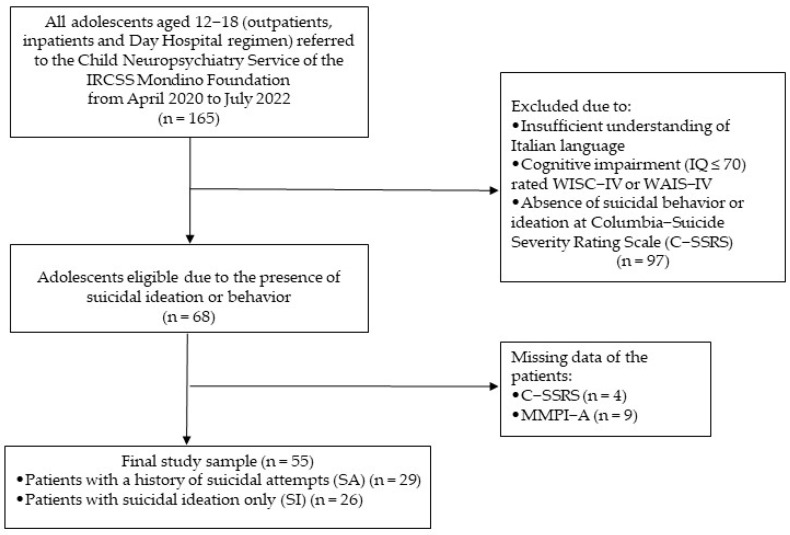
Study population flowchart [author’s own processing].

We recruited 55 adolescents aged 12–18 years (extremes included) from April 2020 to July 2022. All patients who had simply experienced suicidal ideation (SI) or who had a history of non-fatal suicide attempts or interrupted ones (SA) were admitted to the Child Neuropsychiatry unit as either inpatients or outpatients. Using the Columbia Suicide Severity Rating Scale (C-SSRS) [35], we divided the sample into two different groups: SI and SA. Patients with intellectual disabilities or with an insufficient level of comprehension of the Italian language were deemed not eligible for the study. We used the Wechsler Intelligence Scale for Children-Fourth Edition (WISC-IV) [36] or the Wechsler Adult Intelligence Scale-Fourth Edition (WAIS-IV) [37], depending on age, to assess the presence of an intellectual disability. Figure 1 shows the study sample flowchart.

### 2.2. Ethics

This clinical cross-sectional study was realized according to the REporting of studies Conducted using Observational Routinely collected health Data (RECORD) (see Supplementary Material). The Ethics Committee of Policlinico San Matteo in Pavia, Italy, approved this study, which was conducted following the declaration of Helsinki (1964) and its later amendment. Patients gave their consent to participate in the study, and their parents signed the written informed consent. They had the possibility to withdraw from the study at any time without explaining. We anonymized data. The dataset is available in the Zenodo repository [38].

### 2.3. Procedures and Measures

A child neuropsychiatrist collected sociodemographic and anamnestic data of the patients and verified that the patients had no intellectual disability and sufficient comprehension of the Italian language. We administered the Columbia Suicide Severity Rating Scale (C-SSRS) to assess the presence and the severity of SI and/or SA in the last 6 months. The C-SSRS is a semi-structured interview to evaluate the patient’s severity of suicidal ideation. The instrument is very sensitive and permits the detection of the presence of suicidal ideation and suicidal behavior, as well as the frequency and gravity thereof. The suicidal ideation spectrum is taken into account by the scale, which goes from “wish to be dead ‘to’ active suicidal ideation with specific plans, intents, and behaviors”.

Thereafter, a neuropsychiatrist or psychologist compiled the Children Global Assessment Scale (CGAS) [39] to assess the subject’s psychosocial and work-related functioning. The CGAS is a 100-point rating scale composed of a continuum that ranges from mental health (91–100 = Superior functioning) to a serious mental disorder with a high risk of death (1–10 = need constant supervision). In addition, the participants compiled the Minnesota Multiphasic Personality Inventory—Adolescent (MMPI-A) [40], a questionnaire used to assess personality in adolescence. The MMPI-A consists of 478 true–false items and presents different scales:

- six validity scales: Lie (L), Variable Response Inconsistency (VRIN), True Response Inconsistency (TRIN), Infrequency 1(F1), Infrequency 2 (F2), Infrequency (F), Correction (K), Cannot Say (?);
- ten clinical scales: Hypochondria (Hs), Depression (D), Hysteria (Hy), Psychopathic deviation (Pd), Masculinity/Femininity (Mf), Paranoia (Pa), Psychasthenia (Pt), Schizophrenia (Sc), Hypomania (Ma), Social Introversion (Si);
- 15 content scales: Anxiety (A-anx), Obsessiveness (A-obs), Depression (A-dep), Health Concerns (A-hea), Alienation (A-aln), Bizarre Mentation (A-biz), Anger (A-ang), Cynicism (A-cyn), Conduct Problems(A-con), Low Self-Esteem (A-lse), Low Aspirations (A-las), Social Discomfort (A-sod), Family Problems (A-fam), School Problems(A-sch), Negative Treatment Indicators (A-trt);
- six supplementary scales: MacAndrew-Revised (MAC-R), Alcohol/Drug Problem Acknowledgement (ACK), Alcohol/Drug Problem Proneness (PRO), Immaturity (IMM), Anxiety(A), Repression(R).

Filling in the MMPI-A requires a lot of concentration, motivation, and good comprehension skills. Many adolescents compiled the questionnaire with pleasure because they can unveil personal characteristics and at least talk about themselves and their difficulties with clinicians; for many others, the test could be too demanding. To avoid invalid protocols or a lack of motivation, clinicians and psychologists were available for adolescents to help them read the sentences and better explain their meaning (while still adhering to the rules of administration). Moreover, after the assessment, the referring clinician took time to discuss the results with every patient and their expectations for the following treatment.

### 2.4. Statistical Analyses

We determined the sample size with G*Power [41,42] with an effect size of 0.7 and a power of 0.8. Descriptive analyses of the sample, which included demographic and clinical characteristics, were initially performed on the total sample, and subsequently, on the SA and SI groups separately. The analyses included means and standard deviations (SD) for continuous variables and absolute and relative frequencies for categorical variables. A comparison between the two groups was performed with an independent t-test for the numerical variables and with the Chi-square test for the categorical variables. We applied Fisher’s correction as the number of cases was small. Statistical significance was expressed as *p* < 0.05. All statistical analyses were performed with IBM SPSS version 27.0 [43].

## 3. Results

The sample was formed of 55 participants with SI and/or SA. Table 1 shows the sociodemographic and anamnestic data for the total sample and for the two groups. With the exception of risky behaviors (*p* = 0.027) and social interactions (*p* = 0.045), the two groups were homogeneous. A comparison was made between the two groups concerning psychosocial functioning through the CGAS, from which no significant differences emerged (M_SA_ = 48.81, SD_SA_ = 10.77; M_SI_ = 51, SD_SI_ = 10.5). Regarding suicidal ideation, there were significant differences between the two groups (*p* = 0.004) in intensity but not in frequency. Suicidal ideation was more severe in the SA group than in the SI group. Table 2 shows the differences between the SI and SA groups.

### 3.1. Differences between the SI and SA Groups in the Validity Scales of the MMPI-A

We compared SI and SA groups with the number of MMPI-A protocols with 30 or more omissions, thus considered invalid. The analyses showed that all the SI group questionnaires were valid (n = 26), while in the SA group, 4 questionnaires were invalid. However, by performing the Chi-squared test with Fisher’s correction, emerged no significant differences between the two groups (*p* = 0.113). When compared to the SA group, the SI group often responded to fewer questions (M_SI_ = 5.9; M_SA_ = 2.2; *p* < 0.001), and the difference between the groups was statistically significant; results showed a tendency in the SA group to have higher scores on the L (Lie) Scale (*p* = 0.04) compared to the SI group.

### 3.2. Differences between the SI and SA Groups in the Clinical Scales of the MMPI-A

Regarding the clinical scales, we found that the two groups presented statistically significant differences concerning the Masculinity-femininity scale (Mf) (*p* = 0.015). The SI group presented higher scores than the SA group.

### 3.3. Differences between the SI and SA Groups in the Content Scales of the MMPI-A

The results of the analyses revealed a statistically significant difference in two of the content scales of the MMPI-A questionnaire. We found an elevation in the Conduct Problems (CON) Scale (*p* = 0.026) and in the Low Aspirations (LAS) Scale (*p* = 0.028) in subjects belonging to the SA group.

### 3.4. Differences between the SI and SA Groups in the Supplementary Scales of the MMPI-A

The Repression (R) Scale, which has higher scores in the SA group than the SI group (*p* = 0.034), was the only additional scale where a statistically significant difference between the two groups was found.

### 3.5. Difference between the SI and SA Groups about Specific Items of the MMPI-A Causing an Elevation of the D Scale (Depression)

The analyses performed showed that the SI group and the SA group differed significantly in their responses to item 283, “Almost always I wish I was dead” (*p* = 0.003). It emerged that the subjects belonging to the SA group tended to give more affirmative answers to this item, with a mean score of 0.88, in contrast to the SI group, which had a mean score of 0.54.

## 4. Discussion

The present study aimed to investigate whether there were any differences in the validity, clinical, content, and additional scales of the MMPI-A between subjects who had experienced suicidal ideation only (SI) and those who had a history of non-fatal suicidal behaviors (SA). To the best of our knowledge, there are no further studies in the literature that investigate this issue in a sample of adolescents. Qualitative analyses in our study indicated no significant differences between the SI and SA groups in terms of familiarity with psychiatric disorders, diagnoses, and academic achievements. As the two groups presented homogenous features, they were considered comparable. There seems to be an association between suicidal behavior and self-directed life-threatening behaviors, such as self-injury and alcohol/substance abuse. According to earlier research, patients who attempt suicide and exhibit a preponderance of internalizing symptoms may turn to drugs as a coping mechanism for depressive symptoms [16,17,44]. In addition, in our study, we also found scores that may indicate the possibility of a correlation between suicidal behavior and social withdrawal. The analyses of our sample indicated that the SA group had more relational problems and social isolation. Our results confirm the findings of a recent review, which indicated that social isolation may be a predictor of SA in all ages and genders [45]. Adolescents who report a lack of social support and feelings of isolation may self-harm and attempt suicide more frequently. Therefore, we can state that they show a greater tendency to social withdrawal and violent behavior, which is more introjected and directed towards themselves rather than others. Piotti and colleagues have explained this hypothesis, supporting that the tendency of adolescents who experience suicidal behaviors to dissimulate their own suffering exists. Pietropolli Charmet’s colleagues pointed out how our culture is characterized by the achievement of perfection and success, which “does not accept the experiences of failure, limitations, and dependence on others”. In this context, people seem to wear a sort of “mask” that might fail during moments of crisis and expose their own vulnerabilities [46]. As the literature pointed out, in a society focused on achievement and success, the possibility of failure is minimized, and the fear of humiliation is a risk factor for SA [47,48].

These subjects do not manifest their suffering to others, which is instead directed towards themselves. Considering this aspect, Pietropolli Charmet’s theory could explain the tendency of SA adolescents to self-procure physical pain through self-injury, hiding their suffering, and showing their competence to the outside world. Even the use of drugs can lead to long-term physical suffering. If initially the use of substances can be considered as a form of self-care, then it might become abuse, causing abstinence symptoms, leading in some cases to death.

According to the findings of our study on SA teenagers’ propensity to conceal their pain, this group of patients may be more inclined to deny feelings of sadness and show a lack of sincerity towards others concerning their personal internal states. This is in line with the literature that shows low agreement between self-reports and clinician assessments regarding SI [49], with high rates of false positives registered in self-report measures compared to clinical assessment [50]. Moreover, previous studies reported different agreements between the family report and clinical assessment [51]. However, other studies report that participants feel more comfortable disclosing information on suicide-related topics through the relative anonymity of a self-rating scale [52,53].

A final difference emerged in our research between the two groups: the SA group reported lower scores on the masculinity–femininity (Mf) scale than the SI subjects. This difference shows that compared to persons exhibiting only SI, female subjects with SA may be less likely to exhibit stereotypically masculine interests and, thus, a lesser inclination to gender reversal. This result is in contrast to other research that suggested the prevalence of “psycho-sexual misfits” based on high Mf scale scores in the SA group; however, those studies focused on adult populations. [54]. We can also hypothesize that given that almost all the male participants in our study belonged to the SI group and had supra-threshold scores in the Mf scale, this difference could also be due to the unusual presence of stereotypically female interests in our sample males. It would be useful to investigate these aspects within the adolescent population, also considering how the concept of “correspondence” between one’s biological gender and certain personological or behavioral characteristics has changed over time. Furthermore, the evolution of adolescents and their role within modern society should be taken into consideration [55,56].

Finally, concerning the intensity of suicidal ideation, in the present study, the differences in the scores on item 283—“Almost always I wish I was dead”—corroborate the results of several studies which showed that thoughts about death, wishing to be dead, thoughts of suicide, and suicide plans were significantly higher among adolescents with a history of SA [18]. The results from our study are consistent with the literature [18,57], which suggests that a substantial correlation exists between ideation and attempt, and that the existence of profound suicidal ideation and a prior suicidal attempt would be significant predictors of a subsequent attempt at suicide.

## 5. Conclusions

These findings may be useful in clinical practice to identify individuals who are more likely to attempt again; especially it seems important to pay attention to the subject’s relational context. Social isolation may be a factor that increases the presence of suicidal behavior, as well as the presence of self-directed harming behavior. The presence of an externalizing condition is one feature that seems to be less associated with suicide; releasing anger outwardly may lessen the chance of engaging in a self-damaging act. In conclusion, despite the limits of this study due to the presence of a small sample of subjects, the lack of a control group, and the use of a self-administered and long questionnaire, the results of our study encourage the prosecution of research. In fact, at the moment, the small number of participants limits the generalizability of results, but we are enrolling more participants in order to do that. Furthermore, the MMPI-A could be useful in the assessment of adolescents at risk of suicidal behavior, analyzing personality aspects that are supposed to differentiate those who have already made an attempt from those who have not. The study we proposed is innovative, as there are no further studies in the literature that investigate differences in certain aspects of personality using the MMPI-A in a population of adolescents with SI and behavior. However, since this is a preliminary study, it also lacks longitudinal data. So, our research team is enrolling more participants to have a larger sample, continuing to monitor and collect data on the effects that the pandemic has had on this group of people [4,58], looking into potential risk factors for gender dysphoria [56], and including a control group. It may be possible to use the MMPI-A questionnaire as a preventive measure of the risk of suicide attempts in adolescents if it is administered in certain contexts (e.g., schools) together with additional questionnaires that can easily assess the presence of suicidal ideation, such as the Multi-Attitude Suicide Tendency Scale (MAST) [59]. We can hypothesize that by administering this questionnaire, it will be possible to determine which adolescents are most likely to have attempted suicide and to evaluate the presence of these characteristics in subjects who already present SI. This will allow us to consider the possibility of an intervention to prevent the act. We have found that certain personality traits, such as repression and a tendency to lie, are associated with the risk of suicide attempts in adolescents. Moreover, those results may represent the first step for future longitudinal studies investigating the interaction between clinical outcomes and the potentiality of MMPI-A as an efficient screening and preventive instrument.

## Figures and Tables

**Table 1:** Sociodemographic and anamnestic data in the total sample and the two subgroups (author’s own processing).

Variable: Number of Subjects and Percentage Indicated		Total (n = 55)	SI (n = 26)	SA (n = 29)
Gender	Male Female	4 (7.3) 51 (92.7)	3 (11.5) 23 (88.5)	1 (3.5) 28 (96.5)
Ethnicity	Caucasian African Latin (South America) Mixed Asian	47 (85.5) 3 (5.5) 2 (3.6) 2 (3.6) 1 (1.8)	20 (76.9) 2 (7.7) 1 (3.8) 2 (7.7) 1 (3.8)	27 (93.1) 1 (3.6) 1 (3.6) 0 0
Siblings	Only child Major Minor Major and minor	9 (16.4) 15 (27.2) 25 (45.5) 6 (10.9)	4 (15.4) 7 (26.9) 11 (42.3) 4 (15.4)	5 (17.2) 9 (31.1) 13 (44.8) 2 (6.9)
Parents	Married Divorced	31 (58.1) 23 (41.9)	15 (57.7) 11 (42.3)	16 (57.1) 12 (42.9)
SES	Low Medium-low Medium Medium-high High	5 (11.9) 3 (7.1) 13 (30.9) 13 (30.9) 8 (19.2)	4 (23.5) 0 5 (29.4) 5 (29.4) 3 (17.7)	1 (4) 3 (12) 8 (32) 8 (32) 5 (20)
Previous consultations	Absent Present	12 (22.2) 42 (77.8)	5 (19.3) 21 (80.7)	7 (25) 21 (75)
Social relationships	Social withdrawal Poor relations Adequate relations	6 (10.9) 30 (54.5) 19 (34.6)	0 16 (61.5) 10 (38.5)	6 (20.7) 14 (48.3) 9 (31.0)
Familiarity	Depression (sub-threshold) Depression (supra-threshold) Bipolar disorder (sub-threshold) Anxiety (sub-threshold) Substance abuse Eating behavior disorder Opp. Defiant behavior disorder Personality disorder (sub-threshold) Other	19 (34.5) 2 (3.6) 1 (1.8) 9 (16.4) 2 (3.6) 4 (7.3) 1 (1.8) 1 (1.8) 23 (41.8)	9 (34.6) 0 1 (3.8) 4 (15.4) 2 (7.7) 1 (3.8) 1 (3.8) 1 (3.8) 12 (46.2)	10 (34.5) 2 (6.9) 0 5 (17.2) 0 3 (10.3) 0 0 11 (37.9)
Risky behaviors	Substance use, self-harm, SA	40 (72.7)	15 (57.7)	25 (86.2)
Academic performance	Poor Sufficient Good Excellent School withdrawal	9 (16.3) 14 (25.5) 15 (27.7) 12 (21.8) 5 (9.1)	3 (11.5) 5 (19.3) 8 (30.8) 7 (26.9) 3 (11.5)	6 (20.7) 9 (31.1) 7 (24.1) 5 (17.2) 2 (6.9)
Access modes	Outpatient clinic Day hospital Hospitalization	13 (23.6) 1 (1.8) 41 (74.5)	5 (19.3) 1 (3.8) 20 (76.9)	8 (27.6) 0 21 (72.4)
Diagnosis	Neurodevelopmental disorder Schizophrenia/psychotic disorders Bipolar and related Depression Anxiety disorders DOC and related Eating disorders Personality disorders Substance use disorders Other	1 (1.8) 13 (23.6) 2 (3.6) 39 (70.9) 18 (32.7) 3 (5.5) 15 (27.3) 34 (61.8) 1 (1.8) 12 (21.8)	0 4 (15.4) 2 (7.7) 18 (69.2) 10 (38.5) 1 (3.8) 9 (34.6) 7 (26.9) 1 (3.8) 5 (19.2)	1 (3.5) 9 (31.0) 0 21 (72.4) 8 (27.6) 1 (3.5) 6 (20.7) 14 (48.2) 1 (3.5) 7 (24.1)
Previous psychotherapy	Absent Present	31 (56.3) 24 (43.7)	14 (53.8) 12 (46.2)	17 (58.6) 12 (41.4)
Pharmacotherapy prescribed	Absent Present	10 (20.0) 43 (80.0)	6 (23.1) 20 (76.9)	4 (14.8) 23 (85.2)
Type of medication	Antipsychotic Antidepressant Anxiolytic Mood stabilizer	26 (47.2) 20 (36.4) 30 (54.5) 4 (7.3)	9 (32.1) 9 (32.1) 12 (48) 1 (4)	17 (60.7) 11 (39.3) 18 (64.3) 3 (10.7)
CGAS	-	49.9	51	48.81
IQ	-	105.01	104. 52	105.48

**Table 2:** Differences between the SI and SA groups in MMPI-A (author’s own processing).

	SI (n = 26)		SA (n = 25)			
	M	SD	M	SD	t	*p*
Omissions	5.9	5.22	2.2	4.06	2.8	<0.001 **
VRIN	54.2	11.05	55.64	7.55	−0.53	0.299
TRIN-V	58.9	6.11	58.64	8.06	0.103	0.459
F1	72.7	15.68	73.84	15.25	−0.256	0.40
F2	70.5	16.91	75	14.88	−0.998	0.162
F	74.7	16.80	78.12	15.74	0.743	0.231
L	46.3	8.27	50.36	8.50	−1.743	0.044 *
K	45.62	7.45	47.08	6.70	−0.737	0.232
F-K	8.58	10.32	9.04	8.10	−0.178	0.43
Hs	69.92	11.23	68	12.65	0.568	0.286
D	79.88	9.77	79.8	11.49	0.027	0.489
Hy	68.88	11.98	68.88	12.01	0	0.5
Pd	66.60	10.81	68	11.27	−0.448	0.328
Mf	50.52	10.60	44.44	8.46	2.242	0.015 *
Pa	71.72	11.64	72.2	11.31	−0.148	0.442
Pt	71.20	9.67	70.4	10.01	0.287	0.388
Sc	75.72	13.47	73.12	13.43	0.683	0.249
Ma	52.08	8.60	51.68	10.87	0.144	0.443
Si	68.2	8.62	68.68	8.83	−0.195	0.423
ANX	70.4	11.655	72.6	8.836	−0.752	0.228
OBS	61	14.609	60.8	8.912	0.058	0.477
DEP	71.52	10.042	76.52	9.592	−1.8	0.39
HEA	69	11.836	66.8	13.793	0.61	0.274
ALN	73.56	15.314	70.56	12.484	0.759	0.226
BIZ	63.68	12.841	63.64	15.256	0.01	0.496
ANG	55.32	10.152	51.4	8.529	1.478	0.073
CYN	51	11.8	50.56	11.637	0.133	0.447
CON	57.76	15.621	50.52	8.945	2.011	0.026 *
LSE	70.44	14.897	69.88	10.365	0.154	0.439
LAS	66.12	12.862	59.2	12.203	1.951	0.028 *
SOD	75.84	10.703	76.76	13.767	−0.264	0.397
FAM	62.32	13.009	60.6	14.6	0.44	0.331
SCH	64.72	12.023	62.64	11.485	0.625	0.267
TRT	72.68	21.075	70.48	16.83	0.408	0.343
MAC/R	51.08	9.648	51.4	11.188	−0.108	0.457
ACK	65.8	15.538	61.08	16.036	1.057	0.148
PRO	54.68	12.779	53.36	11.608	0.382	0.352
IMM	68.24	13.116	64.8	10.583	1.021	0.156
A	65.08	8.441	66.04	5.827	−0.468	0.321
R	57.64	10.169	62.6	8.602	−1.862	0.034 *
Item 68	0.77	0.43	0.92	0.282	−1.42	0.078
Item 71	0.5	0.51	0.36	0.49	0.999	0.161
Item 124	0.8	0.408	0.92	0.282	−1.159	0.125
Item 177	0.85	0.368	0.84	0.374	0.059	0.477
Item 283	0.54	0.508	0.88	0.332	−2.829	0.003 *
Item 347	0.84	0.374	0.87	0.338	−0.343	0.366
Item 371	0.81	0.402	0.8	0.408	0.068	0.473
Item 372	0.81	0.402	0.76	0.436	0.406	0.343
Item 399	0.77	0.43	0.84	0.374	−0.626	0.267

Significance: * *p* < 0.05, ** *p* < 0.001.

## Data Availability

Data are available upon reasonable request in Zenodo (10.5281/zenodo.7599703).

## Supplementary Materials

The following supporting information can be downloaded at: https://www.mdpi.com/article/10.3390/children10111794/s1. Ref. [60] is cited in the Supplementary Materials.
